# Predicting Progression, Recurrence, and Survival in Pancreatic Neuroendocrine Tumors: A Single Center Analysis of 174 Patients

**DOI:** 10.3389/fendo.2022.925632

**Published:** 2022-06-28

**Authors:** Sara Krogh, Henning Grønbæk, Anders Riegels Knudsen, Peter Kissmeyer-Nielsen, Nynne Emilie Hummelshøj, Gitte Dam

**Affiliations:** ^1^ Department of Hepatology and Gastroenterology, Aarhus University Hospital, Aarhus, Denmark; ^2^ Department of Gastrointestinal Surgery, Aarhus University Hospital, Aarhus, Denmark

**Keywords:** pancreatic neuroendocrine tumor (PNET), prognosis, survival, recurrence, ENETS

## Abstract

**Introduction:**

The European Neuroendocrine Tumor Society, ENETS, reports variables of prognostic significance in pancreatic neuroendocrine tumors (PNET). However, studies have short follow-ups, and the optimal treatment remains controversial. We aimed to determine overall survival (OS), progression-free survival (PFS) after conservative treatment, and recurrence-free survival (RFS) after surgery and further to find predictors of aggressive PNET behavior to support treatment decisions.

**Methods:**

174 patients with PNET treated at Aarhus University Hospital from 2011 to 2021 were included in a retrospective cohort study. Patients were divided into surgically resected (*SUR*, n=91) and medically or conservatively treated (*MED*, n=83). Variables were tested in univariate and multivariate survival analysis. Median follow-up time was 3.4 years in the MED group and 4.5 years in the SUR group.

**Results:**

The 5-year OS was 95% and 65% for the SUR and MED groups, respectively. The 5-year RFS in the SUR group was 80% whereas the 5-year PFS in the MED group was 41%. Larger tumor size, Ki67 index, tumor grade, and stage were predictive of shorter OS, RFS, and PFS. Further, chromogranin A was a predictor of OS. Larger tumor size was associated with higher stage and grade. Only 1 of 28 patients with stage 1 disease and size ≤2 cm developed progression on a watch-and-wait strategy during a median follow-up of 36 months.

**Conclusion:**

This study supported the ENETS staging and grading system to be useful to predict OS, PFS, and RFS in PNET. Further, our data support that small, localized, low-grade PNETS can be followed with active surveillance.

## Introduction

Pancreatic neuroendocrine tumors (PNETs) are rare tumors, constituting 1%-2% of all pancreatic cancers and up to 10% of all NETs (1–3). The incidence has increased in the past 30 years and there has been a significant improvement in survival (3–6). PNETs are classified as functioning (F)- or non-functioning (NF) according to the potential hormone production. NF-PNETs comprise at least 70% and are discovered either incidentally or due to symptoms as a sign of advanced disease (7, 8).

Primary investigation of newly discovered tumors involves staging and grading. The European Neuroendocrine Tumor Society (ENETS) has proposed a staging system, which is widely recognized to predict survival (9). Micro-radical surgery is considered the only curative treatment and is associated with increased overall survival (OS). However, the management is complex, and the benefits of surgery must be weighed against the relatively high risk of perioperative morbidity (3, 10–12). Surgery is considered in early-stage disease but is often contraindicated in widely metastatic disease or in patients with a poor performance status (13, 14).

The incidental detection of asymptomatic PNETs is increasing along with the availability and sensitivity of imaging techniques. These pancreatic incidentalomas (PI) are small and resectable but often have indolent biology. Therefore, well-differentiated PNETs ≤2 cm are often managed conservatively (10, 15). However, approximately 10% of tumors ≤2 cm have lymph node involvement (16, 17), and a meta-analysis suggested a survival benefit for surgery even in smaller tumors (18).

Thus, our study aimed to investigate tumor characteristics in patients with PNETs related to both prognosis and aggressiveness based on the ENETS guidelines. Further, we wished to perform a subgroup analysis of localized PNETS ≤2 cm to test the current guidelines recommending a watch-and-wait strategy in small PNETS (19).

## Methods

**Figure 1:**
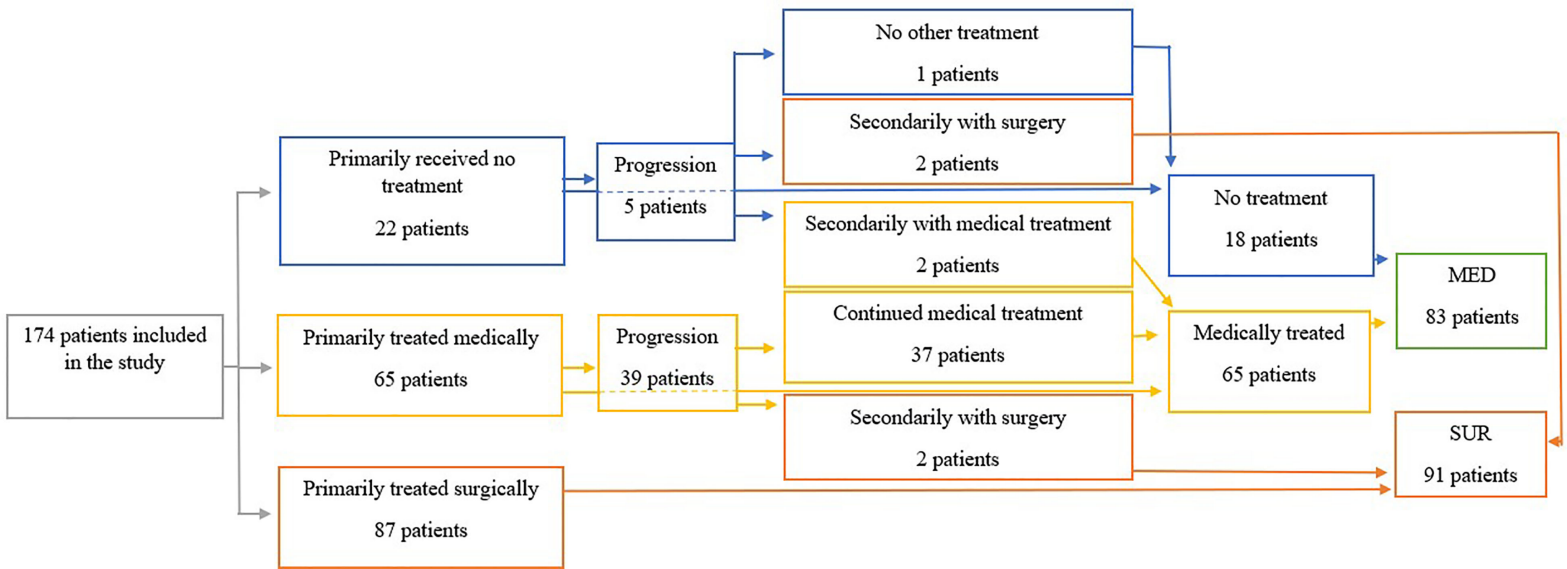
The flowchart of the study. MED, group of patients treated medically or with watchful wait; SUR, surgically treated group.

In this single-center retrospective study, we identified 174 patients with PNET referred to Aarhus ENETS NET center of excellence from 2011 to September 2021. Ninety-one were surgically resected (SUR group) and 83 were medically treated and/or actively followed with a watchful wait strategy (MED group) according to the ENETS guidelines (19). The study was approved as a quality assurance project by The Central Denmark Region Committees on Health Research Ethics. Diagnosis was based on histology or somatostatin receptor-based imaging. See **Figure 1**.

Data were collected from the online record system, Electronic Patient Journal, in 2021 through the unique Civil Personal Registration numbers, given to all Danish citizens and residents (20). It was managed using REDCap electronic data capture tools (21) hosted at Aarhus University.

Patient and tumor characteristics, scan results, pathology, and biochemistry at diagnosis were collected. Tumors were staged according to the ENETS TNM-system (9) and graded based on the Ki67 index at diagnosis into grades 1 (<3%), 2 (3%-20%), and 3 (≥20%) as per 2019 WHO classification (22). Grade 3 comprised both well-differentiated NET-G3 and poorly differentiated neuroendocrine carcinomas, NEC-G3 (10, 11).

### Outcome

Patients were followed until the time of death or until the end of follow-up on September 25, 2021. Median follow-up time was 3.4 years in the MED group and 4.5 years in the SUR group. The primary endpoints were OS, progression-free survival (PFS) in the MED group, and recurrence-free survival (RFS) in the SUR group.

### Statistical Analysis

Data were analyzed using Stata 17 (*StataCorp, College Station, TX USA*). OS was time from diagnosis to death; PFS was time from diagnosis to progression, determined as clinical progression at a multidisciplinary NET tumor board meeting; and RFS was time from surgery to recurrence, or until the end of follow-up. Patients followed for less than 3, 5, and 10 years were censored in the respective survival analysis. OS, PFS, and RFS were calculated with Kaplan-Meier methodology, and log-rank tests compared categorical variables across subgroups. Cox proportional hazard models were used to estimate HR with 95% confidence interval for all significant variables in a univariate analysis and finally in a multivariate analysis to identify independent predictors. Subgroups and association in-between variables were compared using simple t-tests. P-values ≤0.05 were considered statistically significant.

## Results

Of 174 patients, 48% were females and the average age was 65.8 ± 12 and 54.7 ± 13 in the MED and SUR group, respectively. Clinicopathological characteristics are summarized in **Table 1**.

**Table 1:** Clinicopathological data in 174 patients with pancreatic neuroendocrine tumors divided into surgically treated (SUR group, n=91) and medically treated or non-treated (MED group, n = 83).

	MED group*n = 83*	SUR group*n = 91*
**Patients, females, n (%)**	40 (48)	43 (47)
**Age at diagnosis, mean ± SD, Range**	65.8 ± 12 (14;85)	54.7 ± 13* (22;81)
**Charlson score of comorbidities, mean ± SD**	2.3 ± 3	0.9 ± 1*
**F-PNET, n (%)**	6 (7)	20 (22)*
**Insulin**	1 (1)	15 (17)
**Incidentaloma n (%)**	56 (68)	49 (54)
**CgA (pmol/L), mean ± SD**	866.0 ± 3397	153.3 ± 157
**Tumor diameter (cm), mean ± SD**	3.7 ± 4	3 ± 2.4
**Ductus dilatation on scan, n (%)**	14 (17)	9 (10)
**Ki67 index (%), mean ± SD**	12.3 ± 19	10.7 ± 21
**Tumor grade, n (%)**		
**NET-G1 (<3%)**	30 (36)	55 (60)*
**NET-G2 (3-20%)**	22 (27)	20 (22)
**NET-G3 (>20%)**	4 (5)	4 (4)
**NEC-G3**	8 (10)	6 (7)
**Tumor infiltration, n (%)**	17 (21)	9 (10)*
**Lymph node positivity, n (%)**	36 (43)	20 (22)*
**Tumor stage, n (%)**		
**I (T1N0M0)**	31 (37)	42 (46)
**II (T2-3N0M0)**	4 (2)	26 (29)*
**III (T4N0M0/TaN1M0)**	10 (12)	16 (18)
**IV (TaNaM1)**	37 (45)	7 (8)*
**Recurrence/progression, n (%)**	40 (48)	15 (17)*
**Death due to PNET, n(%)**	24 (29)	5 (6)^#^
**Follow-up time (years), mean (range)**	3.4 (0.1-11)	4.5 (0.2-11)

*p ≤ 0.05, statistically significant t-test when comparing the baseline characteristics of the MED group to the SUR group **^#^** significant difference between the equality of survivor function in a log-rank test. CgA, Chromogranin A; F, functioning; NEC, neuroendocrine carcinoma; PNET, pancreatic neuroendocrine tumor.

In the overall MED group, 53% experienced progression and 29% died of PNET. In the SUR group, 16% had recurrence and 6% died of PNET. The 5-year OS was 65% in the MED group versus 95% in the SUR group (p ≤ 0.05). Due to selection bias, the MED and SUR groups are not directly comparable. The MED group had more advanced disease, more co-morbidities, and higher age (**Table 1**).

### Predictors of OS and PFS in the MED Group

The 83 patients in the MED group comprised 20 patients who received no initial treatment. The mean tumor size was 1.3 ± 10 cm. Further, the group comprised 63 patients, medically treated from the time of diagnosis. The mean tumor size was 4.6 ± 4 cm.

**Figure 2:**
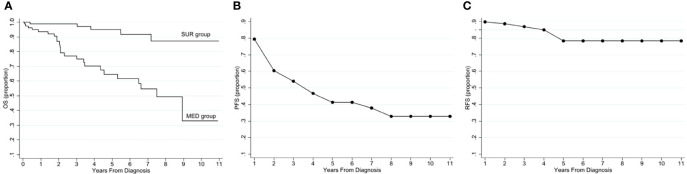
Survival graphs on 174 patients with a primary PNET diagnosis. **(A)** Overall survival in the MED and SUR groups, **(B)** progression-free survival in the MED group, **(C)** recurrence-free survival in the SUR group. MED, group of patients treated medically or with watchful wait; OS, overall survival; PFS, progression-free survival; PNET, pancreatic neuroendocrine tumor; RFS, recurrence-free survival; SUR, group of surgically treated patients.

The 3-, 5-, and 10-year OS was 77%, 65%, and 38%, and the 3-, 5-, and 10-year PFS was 54%, 41%, and 33%, respectively (**Figure 2**).

F-PNET, Chromogranin A (CgA) ≥200pmol/L, high Ki67 index, G3-NEC, lymph node positivity, and tumor stage IV were significant negative predictors of OS (**Tables 2**, **3**). CgA, Ki67 index, and tumor stage IV were also significant in the multivariate analysis (p ≤ 0.05).

**Table 2:** Univariate analysis on determinants of mortality and 5-year survival (%) in 174 patients with pancreatic neuroendocrine tumors divided into surgically treated (SUR group, n = 91) and medically treated or non-treated (MED group, n = 83).

	PFS	OS (MED)	RFS	OS(SUR)
**Overall survival**	41	65	80	95^#^
**Age**				
**<55**	37	68	71	97
**>55**	42	65	90	92
**Sex**				
** Male**	50	68	72	90*
** Female**	31	63	88	100
**Incidentaloma**	54*	74	85	93
** Symptomatic**	22	54	77	96
**F-PNET**	44	33*	75	100
** NF-PNET**	17	70	90	93
**Size**				
**<20mm**	61	83	97	100
**≥20mm**	35*	62	68*	91
**DD**	47	51	60	97
** No DD**	47	73	81	71*
**CgA (pmol/l).**				
**<200**	53	78	75	92
**≥200**	24	50*	67	100
**Grade**				
** NET-G1 (<3)**	56	65	92	100
** NET-G2 (3-20)**	24*	68*	48*	89*
** NET-G3 (>20%)**	14*	100*	60*	100
** NEC-G3**	0*	8*	23*	58*
**Tumor infiltration**	29*	57	37*	100
** Localized**	44	67	84	91
**N0**	77	91	94	97
** N1**	11*	48*	49*	87
**Tumor stage**				
** I**	95	100	98	100
** II**	100	100	79*	92
** III**	52*	70*	43*	93
** IV**	7*	46*	55*	83

*p ≤ 0.05, comparing 5-year survivals within the variable categories in a log-rank test ^#^p ≤ 0.05, comparing the overall 5-year survivals in the MED and SUR groups. CgA, Chromogranin A; DD, ductus dilatation; F, functioning; NEC, neuroendocrine carcinoma; NF, non-functioning; OS, overall survival; PFS, progression-free survival; PNET, pancreatic neuroendocrine tumor; RFS, recurrence-free survival.

**Table 3:** Kaplan-Meier survival analysis in 174 patients with pancreatic neuroendocrine tumors divided into surgically treated (SUR group, n = 91) and medically treated or non-treated (MED group, n = 83).

	OS (MED)	PFS	OS (SUR)	RFS
	HR (95% CI)	HR (95% CI)	HR (95% CI)	HR (95% CI)
**Incidentaloma**		0.3 (0.2;0.6)*		
**F-PNET**	2.8 (1.03;7.7)*			
**Tumor size**				
**≥20mm**		3.1 (1.3;7.3)*		9.0 (1.2;68.7)*
**Ductus dilatation**			12.2 (2.0;73.0)*	
**CgA≥200**	2.9 (1.2;7.4)*			
Ki67 index**	1.02 (1.01;1.03)*	1.03 (1.02;1.05)*	1.1 (1.02;1.08)*	1.02 (1.01;1.04)*
**Grade**				
** NET-G2 (3-20)**	1.2 (0.5;3.2)	3.1 (1.4;6.9)*	3.1 (0.2;50)	9.3 (2.4;36.5)*
** NET-G3 (>20)**		4.6 (1.2;17.8)*	14.5 (0.9;233.5)	5.0 (0.5;48.3)
** NEC-G3 (>20)**	6.5 (2.2;19.2)*	16.0 (5.5;46.3)*	23.3 (2.1;260.6)*	20.4 (4.5;93.3)*
**Tumor infiltration**		2.1 (1.1;4.0)*		3.7 (1.2;11.6)*
**Lymph node involvement**				
** N1**	5.0 (1.7;14.7)*	11.1 (4.5;27.3)*		8.1 (2.4;26.7)*
**Tumor stage**				
** II**	0.0 (-)	0.0 (-)		6.9 (0.8;61.3)
** III**	0.0 (-)	11.4 (1.3;101.9)*		24.8 (3.0;202.5)*
** IV**	8.5e+9 (2.5e+9;2.1e+10)*	48.4 (6.6;355.6)*		22.1 (2.3;212.4)*

*p ≤ 0.05, statistically significant Hazard Ratio when comparing within categories **continuous variable. CgA, Chromogranin A; CI, confidence interval; F, functioning; HR, hazard ratio; NEC, neuroendocrine carcinoma; NF, non-functioning; OS, overall survival; PFS, progression-free survival; PNET, pancreatic neuroendocrine tumor; RFS, recurrence free survival.

Incidental discovery, size ≥ 2 cm, a higher Ki67 index, G2/G3-NET, G3-NEC, local infiltration, lymph node positivity, and tumor stages III and IV were significant negative predictors of PFS (**Tables 2**, **3**). In the multivariate analysis, tumor stage IV, Ki67 index, and G3-NEC were significant (p ≤ 0.05).

### Predictors of OS and RFS in the SUR Group

In the SUR group, the 3-, 5-, and 10-year OS were 99%, 95%, and 87%, and the 3-, 5-, and 10-year RFS was 85%, 80%, and 80%, respectively (**Figure 2**).

Ductus dilatation, high Ki67 index, and G3-NEC were significant negative predictors of OS (**Tables 2**, **3**). In the multivariate analysis, G3-NEC was significant (p ≤ 0.05).

Size ≥ 2 cm, Ki67 index, G2-NET, G3-NEC, local infiltration, lymph node positivity, and tumor stages III and IV were significant negative predictors of RFS (**Tables 2**, **3**). In the multivariate analysis, tumor stage IV and lymph node positivity remained significant (p ≤ 0.05).

### Correlation Between Tumor Size, Stage, and Grade

Larger tumor size was related to lymph node positivity, metastatic disease, and higher grade (p ≤ 0.05, **Table 4**).

**Table 4:** The significance of mean tumor size on tumor grade and stage in pancreatic neuroendocrine tumors.

	Mean size (cm)
**Lymph node positive**	5
**Lymph node negative**	2.4*
**Stage IV**	4.9
**Stage I-III**	2.9*
**G3**	5.9
**G1/G2**	3.5*
**Infiltration**	6.9
**Local**	2.7*

*p ≤ 0.05, significant difference in size when comparing predictors of aggressive tumor behavior.

### Subgroups

The frequency of lymph node positivity was 25% in PI versus 47% in symptomatic tumors (p ≤ 0.05). The mean Ki67 index was 8% in PI and 16% in symptomatic tumors (p ≤ 0.05). No other variables differed in the two groups. Overall, the F- and NF-PNET groups were identical, except that the patients with F-PNET were younger at diagnosis (54 years) compared to patients with NF-PNET (61 years) (p ≤ 0.05).

### Insulinomas and Repeated Analysis After Exclusion

The SUR group comprised 15 insulinomas, and among these, there were no deaths. Only one patient experienced progression. Insulinomas have an excellent prognosis and all analyses were repeated after exclusion of these. Ki67 index and NEC-G3 were still significant predictors of survival in the non-insulinoma SUR group (p ≤ 0.05). Ki67, G1- and NEC-G3, tumor infiltration, lymph node positivity, and tumor stage were still predictors of recurrence (p ≤ 0.05). The 5-year OS and RFS in the non-insulinoma SUR group were reduced to 94% and 76%, respectively.

### Tumors ≤2 cm

Fifty-nine patients with tumors ≤2 cm had localized G1 disease; 31 in the SUR group and 28 in the MED group. All 28 patients in the MED group were followed with a watch-and-wait strategy, and the group comprised 16 patients who were followed without treatment and 12 patients who were followed on Somatostatin Analogues. During a median follow-up of 36 months only one in 28 patients in the MED group experienced progression while 1 in 31 patients in the SUR group experienced recurrence during a median follow-up of 56.5 months. The SUR and MED groups differed in age, PI, and F-PNET (**Table 5**).

**Table 5:** Clinicopathological data in 59 patients with stage I pancreatic neuroendocrine tumors ≤2 cm divided into surgically treated (SUR group, n = 31) and medically treated or non-treated (MED group, n = 28).

	MED group *n = 28*	SUR group *n = 31*
**Gender, female, n (%)**	16 (57)	12 (39)
**Age at diagnosis, mean ± SD**	67 ± 14	57 ± 10*
**Incidentaloma n (%)**	26 (93)	16 (52)*
**F-PNET, n (%)**	0 (0)	13 (41)*
**Tumor diameter (cm), mean ± SD**	1.3 ± 0.4	1.4 ± 0.5
**Ki67 index (%), mean ± SD**	1.1 ± 0.3	0.9 ± 0.5
**CgA (pmol/L), mean ± SD**	205 ± 372	148 ± 203
**Recurrence/progression, n (%)**	1 (3)	1 (3)
**Death due to PNET, n (%)**	1 (3)	1 (3)

*p ≤ 0.05, statistically significant t-test when comparing the baseline characteristics of the MED group to the SUR group. CgA, Chromogranin A; F, functioning; PNET, pancreatic neuroendocrine tumor.

## Discussion

This large single-center cross-sectional study from an ENETS center of excellence demonstrated that high TNM-stage is a significant predictor of both RFS in surgically treated patients and PFS and OS in patients treated medically or with no treatment. This is in agreement with previous studies demonstrating that stage IV disease is the strongest predictor of a poor prognosis regardless of any other variable (23–25). We further demonstrated that high tumor grade is a strong, negative predictor of OS. Both tumor stage and grade are widely used for prognostic assessment, and the ENETS classification system for PNET has been evaluated previously (23, 25–29). In line with Ekeblad et al. and Scarpa et al. (23, 26), we found no significant difference between stage I and II disease (**Table 2**). This was also the case for G1 and G2 tumors and this may be caused by type 2 error. Meanwhile, Ki67 was a significant predictor of OS both in the MED and in the SUR groups. This is supported by Panzuto et al. (30) who also found that an increase in the Ki67 index was associated with poorer survival.

Brooks et al. showed that surgery in PNET is an independent predictor of OS (31). Our findings support these data as we demonstrated a higher 5-year OS after surgery (95% versus 65%). However, our study is non-randomized and retrospective, and the selection of the patients biases our results. Surgery was performed in younger patients with lower grade and stage tumors and results should be interpreted with caution and with this selection bias in mind. F- and NF-PNETs are suggested to differ in aggressiveness and hence have a different prognosis (7, 23, 25, 26, 28). We were unable to demonstrate this. Insulinomas have an excellent prognosis after surgery, and we therefore, tried to exclude these and repeat all analyses. The 5-year OS remained excellent in the SUR group and only minor changes in the RFS were observed.

A recent meta-analysis demonstrated that post-surgery recurrence was higher in patients with high grade tumors, lymph node involvement, or vascular invasion (32). This is in accordance with our study, demonstrating that patients with higher stage and grade have a decreased RFS after surgery and therefore warrant closer follow-up.

In agreement with previous literature, PIs had a more indolent behavior compared to those that were symptomatic at diagnosis. The PFS in our study was longer, and they were more likely to be lymph node negative and have a low grade. This coheres with the fact that they are discovered early (8, 10, 16, 17, 24).

### Tumors ≤2 cm

The 2016 ENETS Consensus Guidelines (19) suggest a conservative approach in non-metastatic, NF-tumors ≤2 cm. Our findings support that tumor size is a predictor of aggressive behavior. We demonstrated that larger tumor size was predictive of both higher stage and grade but also the presence of lymph node metastasis. Smaller size was also associated with lower RFS after surgery (**Table 3**). This all agrees with previous studies (23, 25–27, 32).

Betinni et al. found that tumors ≤2 cm predicted a non-indolent behavior and therefore advocated against surgery (29). Kuo et al. and Haynes et al. put this into perspective and demonstrated that the natural history is variable and the course difficult to predict (4, 16). Overall, they showed that PI can display aggressive behavior despite small size. Further, a meta-analysis from 2017 demonstrated survival benefits in tumors ≤2 cm (18).

In our study, 59 patients had stage I tumors ≤2 cm and 53% underwent surgery. The SUR group comprised more functioning tumors and younger patients than the MED group. Only 3% of the resected tumors ≤2 cm showed recurrence. This supports a recent study from Sallinen et al. who demonstrated an excellent disease-free survival after surgery (33). As long-term results after surgery are excellent, the outcome of non-operative management of tumors ≤2 cm is of paramount interest. The 28 patients in the MED group were followed with a watch-and-wait strategy. Only one patient (3%) experienced progression. Although the total number is limited, our findings support a conservative approach in accordance with the 2016 ENETS Consensus Guidelines (19).

In conclusion, in this large cohort of PNETs we demonstrated that high TNM-stage, tumor grade, Ki67 index, size, CgA, and symptomatic discovery are negative prognostic predictors of survival. Further, the surgically treated group had the highest survival, and we support the guidelines recommending surgery when predictors of aggressive tumor behavior are present. Further, we believe that a watch-and-wait strategy with active surveillance can be followed in patients with low grade, low stage NF-PNET ≤2 cm (19).

## Data Availability Statement

The raw data supporting the conclusions of this article will be made available by the authors, without undue reservation.

## Author Contributions

SK: conceptualization, methodology, formal analysis, investigation, resources, writing – original draft, writing – review and editing. GD: conceptualization, methodology, writing – review and editing, supervision, project administration. HG: conceptualization, methodology, writing – review and editing, supervision. NH: writing – review and editing. PK-N: methodology, writing – review and editing. AK: methodology, writing – review and editing. All authors contributed to the article and approved the submitted version.

## Conflict of Interest

HG received research funding from Intercept, Abbvie, NOVO Nordisk Foundation, Arla, and ADS AIPHIA Development Services AG. Advisory board at Ipsen and Pfizer. Speaker Norgine Takeda.

GD received research funding from IPSEN, AAA.

## Publisher’s Note

All claims expressed in this article are solely those of the authors and do not necessarily represent those of their affiliated organizations, or those of the publisher, the editors and the reviewers. Any product that may be evaluated in this article, or claim that may be made by its manufacturer, is not guaranteed or endorsed by the publisher.
